# A Rare Case of Extramedullary Relapse of Multiple Myeloma Causing Bowel Obstruction

**DOI:** 10.1155/2019/4962678

**Published:** 2019-04-11

**Authors:** Prarthna V. Bhardwaj, Shrinkhala Khanna, Majd D. Jawad, Syed S. Ali

**Affiliations:** ^1^Resident, Department of Internal Medicine, Baystate Medical Center, Springfield, MA, USA; ^2^Assistant Professor, Department of Hematology/Oncology, UMass Medical Center, Worcester, MA, USA; ^3^Resident, Department of Pathology, Baystate Medical Center, Springfield, MA, USA; ^4^Assistant Professor, Department of Hematology Oncology, Baystate Medical Center, Springfield, MA, USA

## Abstract

Extramedullary myeloma, defined by presence of plasma cells outside the bone marrow, is a rare entity accounting for about 3–9% of all cases. It usually is aggressive with a median survival of <6 months. It is also associated with adverse prognostic factors including 17p deletions and high-risk gene profiles. While common extramedullary sites include bones, there have been several case reports of hematogenous extramedullary myeloma to the liver, lungs, pancreas, breast, skin, and soft tissues. Extramedullary myeloma to the mesentery is a rare entity with only a handful of cases reported. We present a case of 69-year-old man presenting with relapse of multiple myeloma to the mesentery, resulting in bowel obstruction to highlight the various presentations of myeloma.

## 1. Introduction

Multiple myeloma is characterized by malignant proliferation of plasma cells that usually produce a unique monoclonal immunoglobulin or parts of it. Although it is an incurable disease, there have been several advances in treatment over the past two decades that have greatly improved the overall survival.

The most common presentation of multiple myeloma is clonal expansion in the bone marrow. Roughly 3–9% of patients have pathogenic clonal plasma cell infiltrates that can be found at various anatomical sites, such as the liver, kidney, breasts, testes, skin, lungs, bones, and other tissues which is called extramedullary disease [1, 2].

Clinically, extramedullary disease can be characterized as follows: tumor masses adjacent to bone and extending into soft tissues; soft tissue or visceral tumors that are not connected to the bone; or diffuse infiltration by plasma cells without any obvious focal lesion [2, 3]. Extramedullary disease tends to have an unfavorable prognosis with a significantly shorter overall survival, even in the era of novel agents [4].

Mesenteric myeloma is a very rare condition with about 12 case reports on the same [5–11]. Here, we present a case of extramedullary myeloma relapse, resulting in bowel obstruction which is a very rare entity and highlights its varied presentation.

## 2. Case Presentation

A 69-year-old Caucasian male was referred to our hospital with 3 weeks of abdominal distension and worsening right lower quadrant pain. He was diagnosed with IgG kappa multiple myeloma four years prior to presentation. He was initially treated with bortezomib/dexamethasone with monthly zolendronic acid with good response initially; however, a year after diagnosis, he was found to have disease progression which manifested as a right radius fracture. His regimen was switched to lenalidomide with dexamethasone with good response and clinically depressed levels of paraproteins. After completion of 9 months of therapy, he underwent autologous stem cell transplant with high-dose melphalan. 7 months after bone marrow transplant, his disease progressed with involvement of pericardial fluid. Salvage therapy was initiated with pomalidomide, bortezomib, and dexamethasone which was discontinued a year later due to peripheral neuropathy; however, at the end of treatment, there was no evidence of ongoing disease.

**Figure 1:**
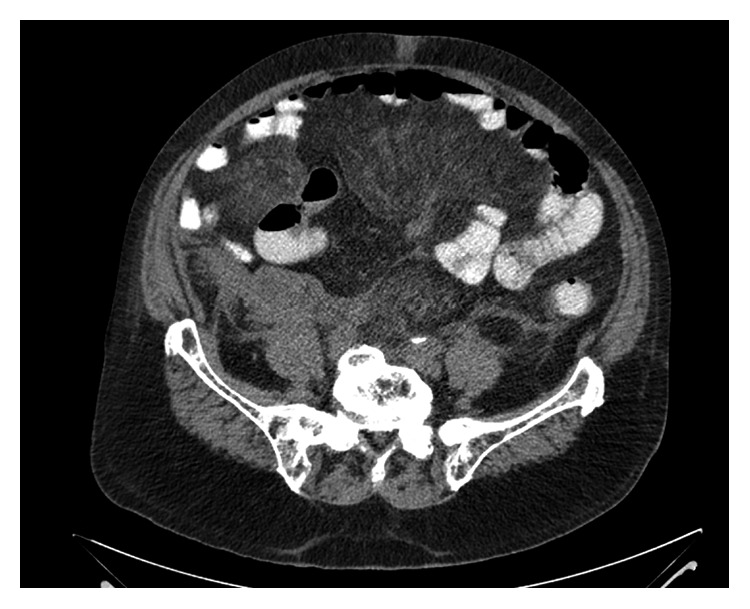
CT scan of the abdomen without contrast revealing mesenteric thickening with edema.

When the patient presented to our hospital, he had an acute abdomen. Initial blood work revealed a normocytic anemia with hemoglobin of 8.4 g/dl and elevated ESR of 44. He also had acute kidney injury with creatinine of 3 mg/dl (baseline of 1.9 mg/dl). CT scan of the abdomen and pelvis revealed extensive stranding seen throughout the abdomen within the peritoneal space with edema in the mesentery (Figure 1).

**Figure 2:**
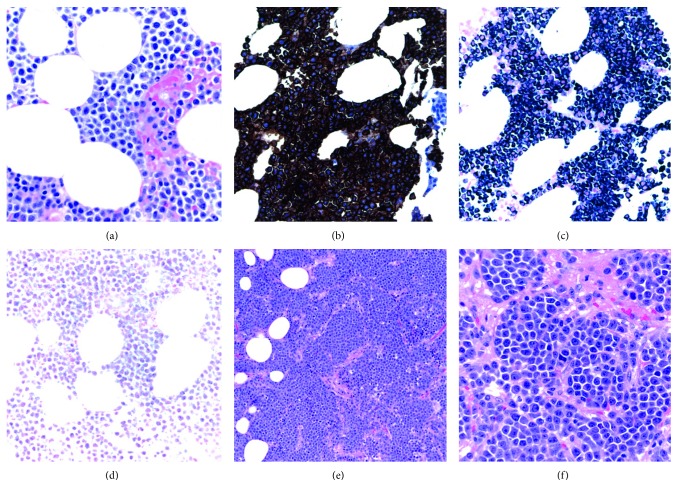
(a) Bone marrow infiltrated by plasma cells. (b) Bone marrow immunohistochemistry revealing CD138 positive cells. (c) Bone marrow immunohistochemistry positive for kappa light chains. (d) Bone marrow immunohistochemistry negative for lambda light chains. Mesenteric invasion (e) by myeloma cells (10x) and (f) by plasma cells (40x).

**Figure 3:**
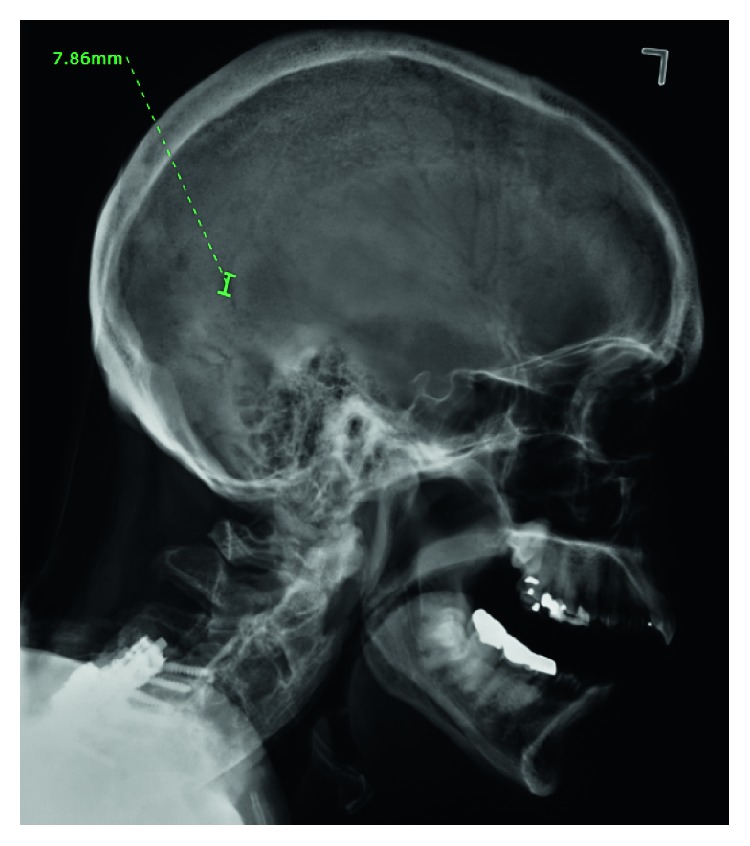
Lytic lesions on the skull.

**Figure 4:**
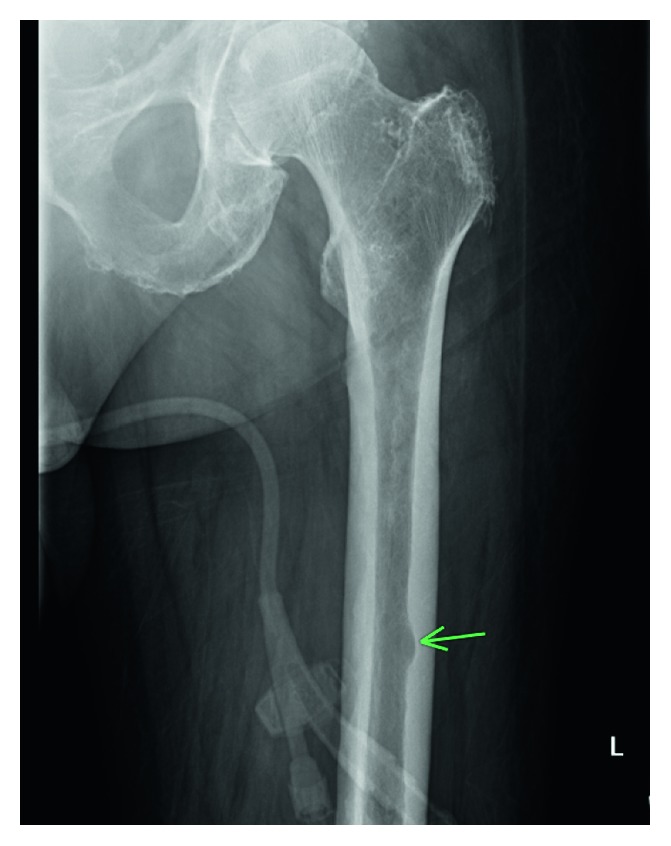
Lytic lesions on the L femur.

He underwent an exploratory laparotomy which revealed induration of the entire base of the mesentery and retroperitoneum. He had an IgG level of 4407 units with predominantly kappa light chains whose level was 4833 units (kappa to lambda ratio 540). Pathology revealed extensive mesenteric infiltration by kappa restricted plasma cells positive for CD138 on immunohistochemistry, without evidence of amyloidosis. Bone marrow biopsy revealed a 30% involvement by plasma cells (Figures 2(a)–2(f)). Cytogenetics showed 1q22 duplication, trisomy 7 and 15, and gain of 8q24.1. The skeletal survey revealed lytic lesions in the left femur and skull (Figures 3 and 4).

He was started on carfilzomib and dexamethasone therapy for relapsed multiple myeloma. Unfortunately, he died within one day of start of the chemotherapy from surgical complications of bowel obstruction.

## 3. Discussion

Extramedullary disease is common at the time of relapse in multiple myeloma. At the time of relapse, there is no rationale to favor a specific therapeutic class of drugs. Previous lines of therapy and the duration of response should clearly be considered. We treated with carfilzomib (CFZ), a second-generation proteasome inhibitor. Stewart et al. in their study noted that the progression-free survival (PFS) was significantly improved with CFZ (median, 26.3 months vs. 17.6 months in control group) especially among patients previously treated with bortezomib and lenalidomide and in patients with high-risk cytogenetics (defined by the presence of *t* (4; 14) and *t* (14; 16) or deletion 17p) [12].

Our case is the one of few case reports of a patient presenting with an abdominal catastrophe from relapsed multiple myeloma, causing tethering of the bowel loops due to plasma cell infiltration in the peritoneum.

As patients live longer with myeloma, multiple relapses may occur from different subclones of plasma cells, and this may be the reason for the more frequent occurrence of extramedullary relapse observed today. PET/CT may have role in close follow-up of patients with extramedullary symptoms.

In conclusion, although extramedullary plasmacytomas occur mainly in the upper aerodigestive tract, mesentery may also be involved with very few case reports in the current literature. The sound combination of radiological, laboratory, and histopathological workup leads to the correct diagnosis for a disease with diverse presentations.
